# Supplementary material for: Genome Sequencing of five Lacticaseibacillus Strains and Analysis of Type I and II Toxin-Antitoxin System Distribution
Source: Microorganisms. 2021 Mar 21;9(3):648. doi: 10.3390/microorganisms9030648 (PMC8003834; doi:10.3390/microorganisms9030648)
Supplement: Supplementary file 1 [file microorganisms-09-00648-s001.zip › microorganisms-1130680 Suppl final/Supplementary figure 1.pptx]

## Slide 1
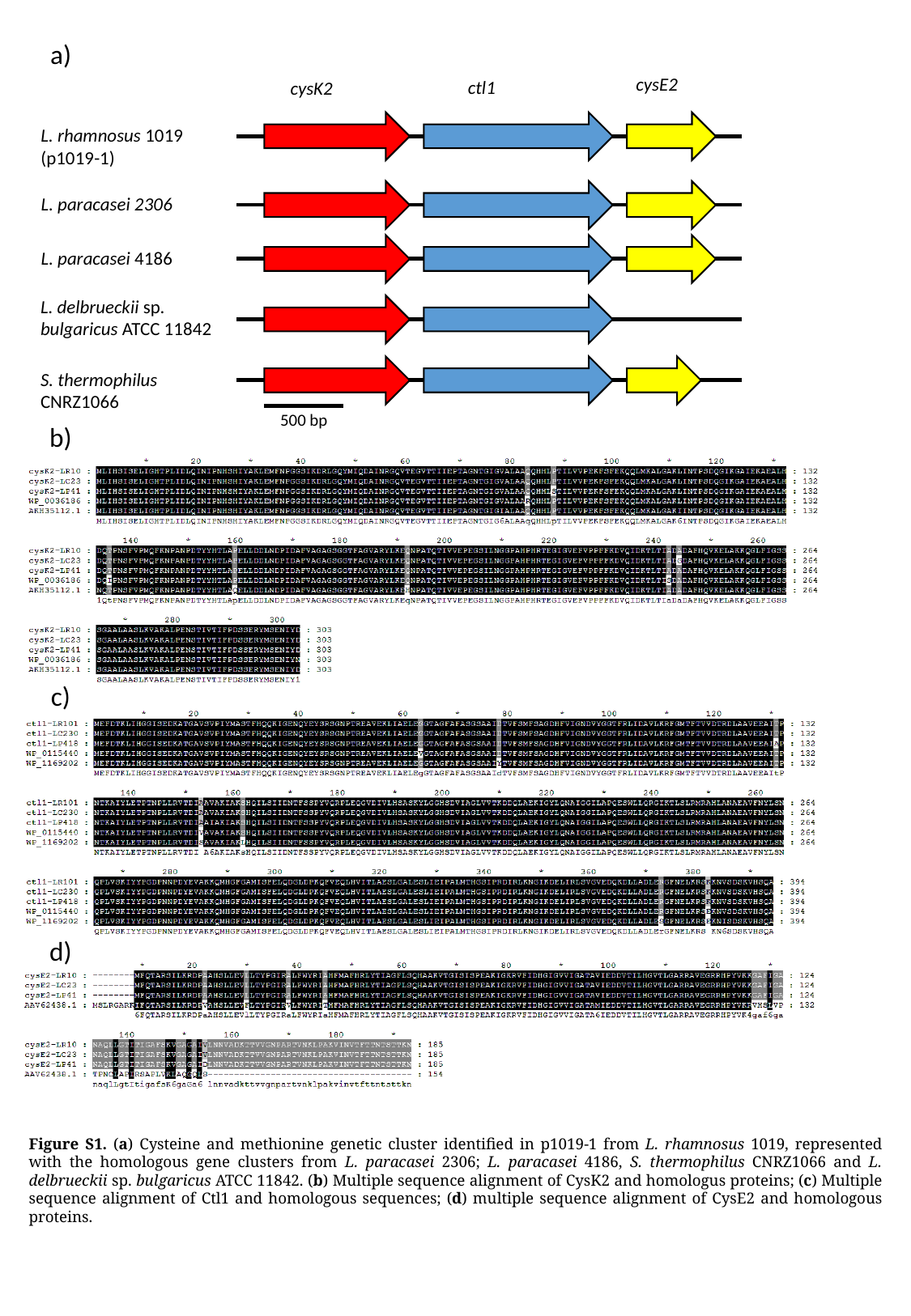

a)
cysE2
ctl1
cysK2
L. rhamnosus 1019
(p1019-1)
L. paracasei 2306
L. paracasei 4186
L. delbrueckii sp. bulgaricus ATCC 11842
S. thermophilus CNRZ1066
500 bp
b)
c)
d)
Figure S1. (a) Cysteine and methionine genetic cluster identified in p1019-1 from L. rhamnosus 1019, represented with the homologous gene clusters from L. paracasei 2306; L. paracasei 4186, S. thermophilus CNRZ1066 and L. delbrueckii sp. bulgaricus ATCC 11842. (b) Multiple sequence alignment of CysK2 and homologus proteins; (c) Multiple sequence alignment of Ctl1 and homologous sequences; (d) multiple sequence alignment of CysE2 and homologous proteins.
