# Supplementary material for: Genome Sequencing of five Lacticaseibacillus Strains and Analysis of Type I and II Toxin-Antitoxin System Distribution
Source: Microorganisms. 2021 Mar 21;9(3):648. doi: 10.3390/microorganisms9030648 (PMC8003834; doi:10.3390/microorganisms9030648)
Supplement: Supplementary file 1 [file microorganisms-09-00648-s001.zip › microorganisms-1130680 Suppl final/supplementary figure 2_rev.pptx]

## Slide 1
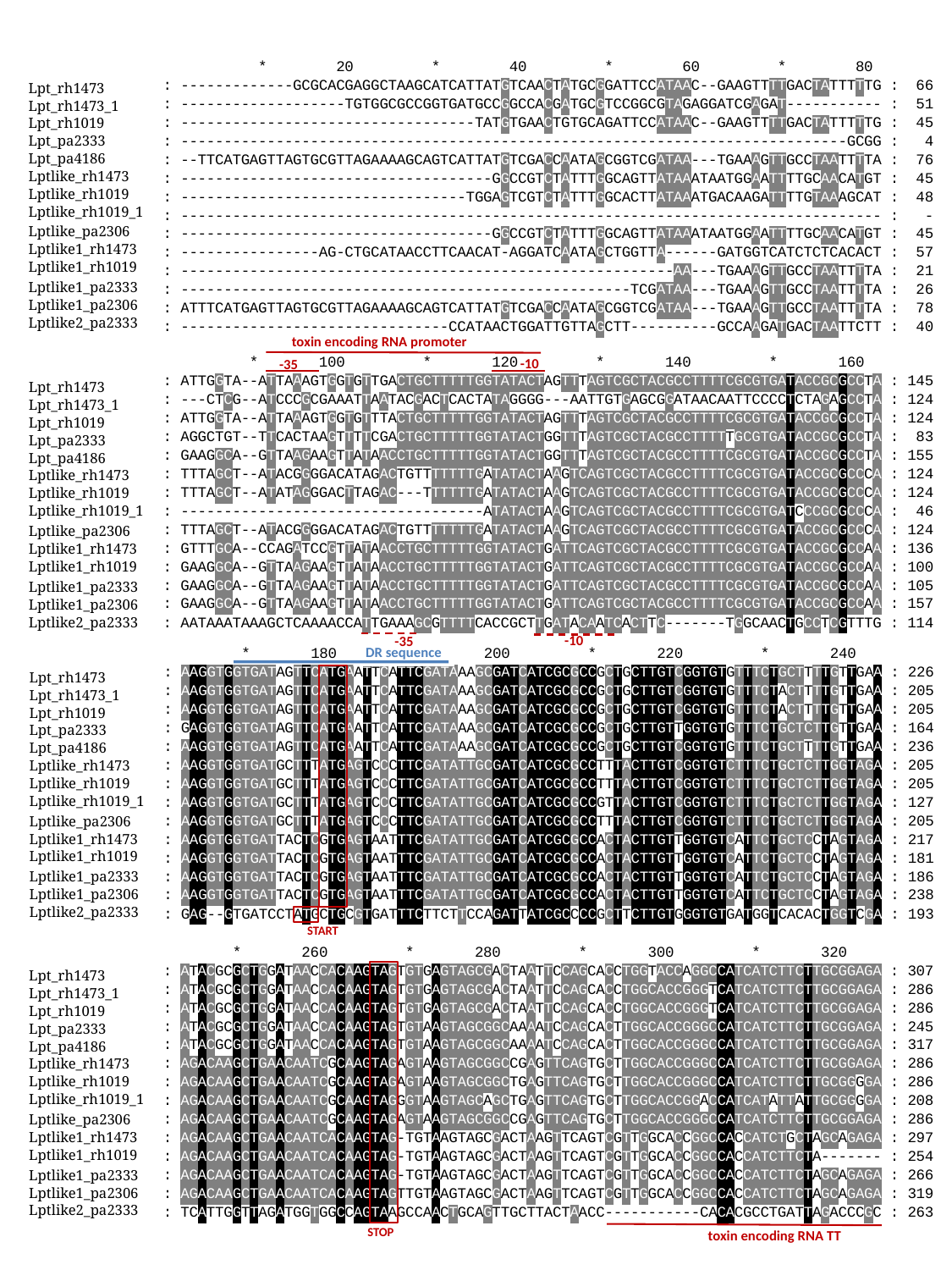

Lpt_rh1473
Lpt_rh1473_1
Lpt_rh1019
Lpt_pa2333
Lpt_pa4186
Lptlike_rh1473
Lptlike_rh1019
Lptlike_rh1019_1
Lptlike_pa2306
Lptlike1_rh1473
Lptlike1_rh1019
Lptlike1_pa2333
Lptlike1_pa2306
Lptlike2_pa2333
toxin encoding RNA promoter
-10
-35
Lpt_rh1473
Lpt_rh1473_1
Lpt_rh1019
Lpt_pa2333
Lpt_pa4186
Lptlike_rh1473
Lptlike_rh1019
Lptlike_rh1019_1
Lptlike_pa2306
Lptlike1_rh1473
Lptlike1_rh1019
Lptlike1_pa2333
Lptlike1_pa2306
Lptlike2_pa2333
-10
-35
DR sequence
START
Lpt_rh1473
Lpt_rh1473_1
Lpt_rh1019
Lpt_pa2333
Lpt_pa4186
Lptlike_rh1473
Lptlike_rh1019
Lptlike_rh1019_1
Lptlike_pa2306
Lptlike1_rh1473
Lptlike1_rh1019
Lptlike1_pa2333
Lptlike1_pa2306
Lptlike2_pa2333
STOP
Lpt_rh1473
Lpt_rh1473_1
Lpt_rh1019
Lpt_pa2333
Lpt_pa4186
Lptlike_rh1473
Lptlike_rh1019
Lptlike_rh1019_1
Lptlike_pa2306
Lptlike1_rh1473
Lptlike1_rh1019
Lptlike1_pa2333
Lptlike1_pa2306
Lptlike2_pa2333
Lpt_rh1473
Lpt_rh1473_1
Lpt_rh1019
Lpt_pa2333
Lpt_pa4186
Lptlike_rh1473
Lptlike_rh1019
Lptlike_rh1019_1
Lptlike_ca2306
Lptlike1_rh1473
Lptlike1_rh1019
Lptlike1_pa2333
Lptlike1_ca2306
Lptlike2_pa2333
toxin encoding RNA TT

## Slide 2
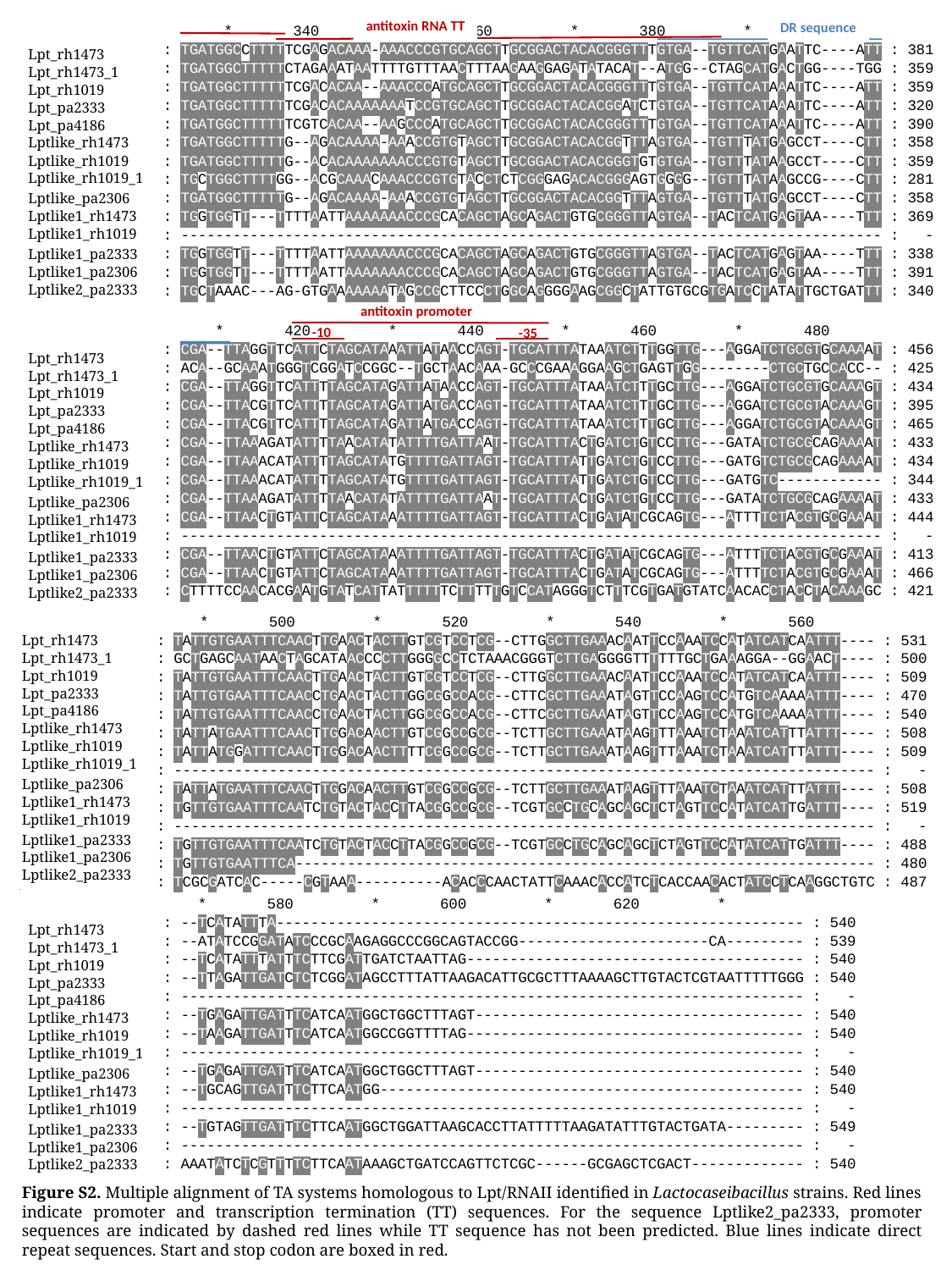

antitoxin RNA TT
DR sequence
Lpt_rh1473
Lpt_rh1473_1
Lpt_rh1019
Lpt_pa2333
Lpt_pa4186
Lptlike_rh1473
Lptlike_rh1019
Lptlike_rh1019_1
Lptlike_pa2306
Lptlike1_rh1473
Lptlike1_rh1019
Lptlike1_pa2333
Lptlike1_pa2306
Lptlike2_pa2333
Lpt_rh1473
Lpt_rh1473_1
Lpt_rh1019
Lpt_pa2333
Lpt_pa4186
Lptlike_rh1473
Lptlike_rh1019
Lptlike_rh1019_1
Lptlike_ca2306
Lptlike1_rh1473
Lptlike1_rh1019
Lptlike1_pa2333
Lptlike1_ca2306
Lptlike2_pa2333
antitoxin promoter
-10
-35
Lpt_rh1473
Lpt_rh1473_1
Lpt_rh1019
Lpt_pa2333
Lpt_pa4186
Lptlike_rh1473
Lptlike_rh1019
Lptlike_rh1019_1
Lptlike_pa2306
Lptlike1_rh1473
Lptlike1_rh1019
Lptlike1_pa2333
Lptlike1_pa2306
Lptlike2_pa2333
Lpt_rh1473
Lpt_rh1473_1
Lpt_rh1019
Lpt_pa2333
Lpt_pa4186
Lptlike_rh1473
Lptlike_rh1019
Lptlike_rh1019_1
Lptlike_ca2306
Lptlike1_rh1473
Lptlike1_rh1019
Lptlike1_pa2333
Lptlike1_ca2306
Lptlike2_pa2333
Lpt_rh1473
Lpt_rh1473_1
Lpt_rh1019
Lpt_pa2333
Lpt_pa4186
Lptlike_rh1473
Lptlike_rh1019
Lptlike_rh1019_1
Lptlike_pa2306
Lptlike1_rh1473
Lptlike1_rh1019
Lptlike1_pa2333
Lptlike1_pa2306
Lptlike2_pa2333
Lpt_rh1473
Lpt_rh1473_1
Lpt_rh1019
Lpt_pa2333
Lpt_pa4186
Lptlike_rh1473
Lptlike_rh1019
Lptlike_rh1019_1
Lptlike_pa2306
Lptlike1_rh1473
Lptlike1_rh1019
Lptlike1_pa2333
Lptlike1_pa2306
Lptlike2_pa2333
Figure S2. Multiple alignment of TA systems homologous to Lpt/RNAII identified in Lactocaseibacillus strains. Red lines indicate promoter and transcription termination (TT) sequences. For the sequence Lptlike2_pa2333, promoter sequences are indicated by dashed red lines while TT sequence has not been predicted. Blue lines indicate direct repeat sequences. Start and stop codon are boxed in red.
