# Supplementary material for: Genome Sequencing of five Lacticaseibacillus Strains and Analysis of Type I and II Toxin-Antitoxin System Distribution
Source: Microorganisms. 2021 Mar 21;9(3):648. doi: 10.3390/microorganisms9030648 (PMC8003834; doi:10.3390/microorganisms9030648)
Supplement: Supplementary file 1 [file microorganisms-09-00648-s001.zip › microorganisms-1130680 Suppl final/supplementary figure 3_rev.pptx]

## Slide 1
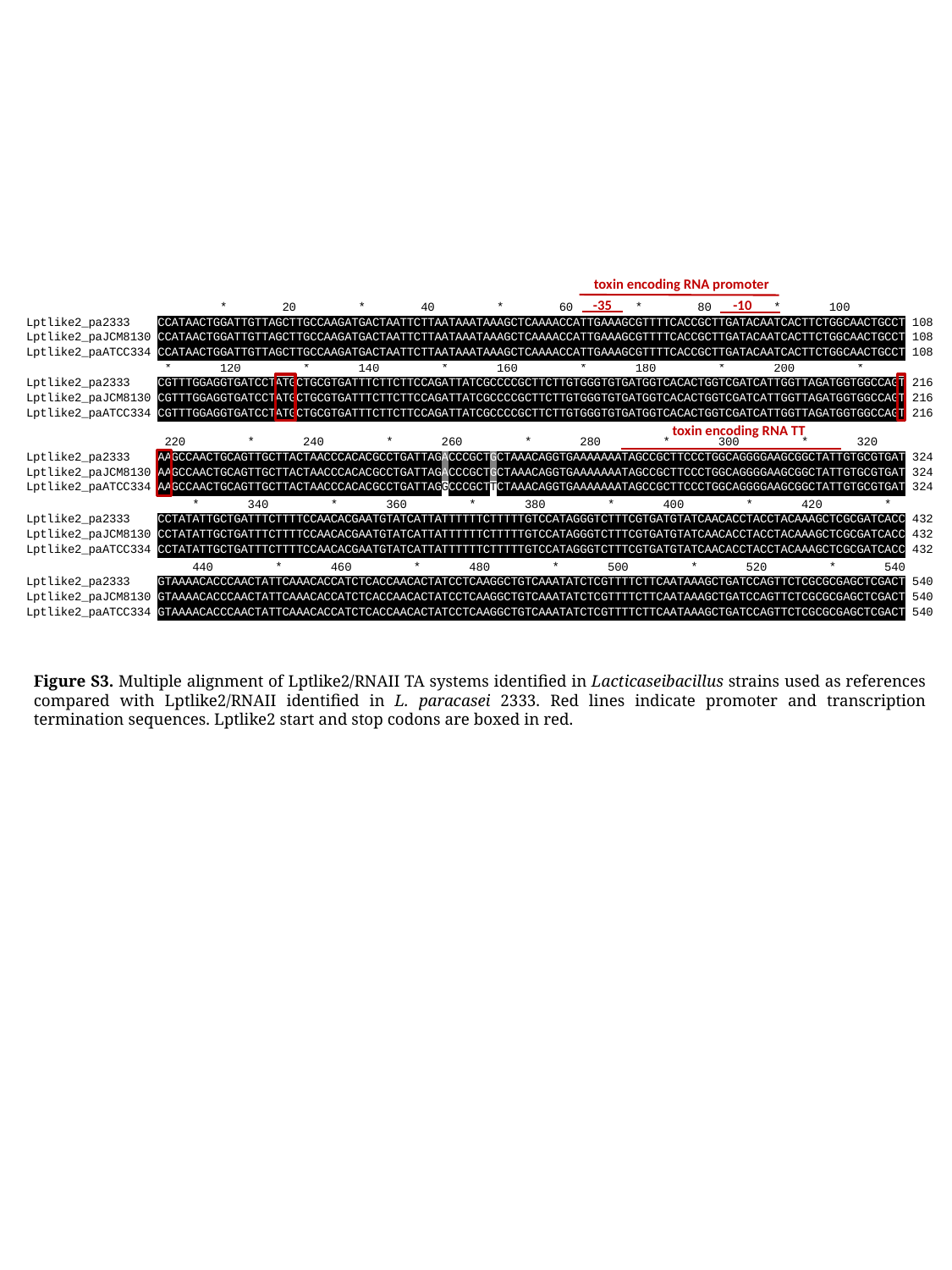

toxin encoding RNA promoter
-35
-10
toxin encoding RNA TT
Figure S3. Multiple alignment of Lptlike2/RNAII TA systems identified in Lacticaseibacillus strains used as references compared with Lptlike2/RNAII identified in L. paracasei 2333. Red lines indicate promoter and transcription termination sequences. Lptlike2 start and stop codons are boxed in red.
