# Supplementary material for: Genome Sequencing of five Lacticaseibacillus Strains and Analysis of Type I and II Toxin-Antitoxin System Distribution
Source: Microorganisms. 2021 Mar 21;9(3):648. doi: 10.3390/microorganisms9030648 (PMC8003834; doi:10.3390/microorganisms9030648)
Supplement: Supplementary file 1 [file microorganisms-09-00648-s001.zip › microorganisms-1130680 Suppl final/supplementary figure 4_rev.pptx]

## Slide 1
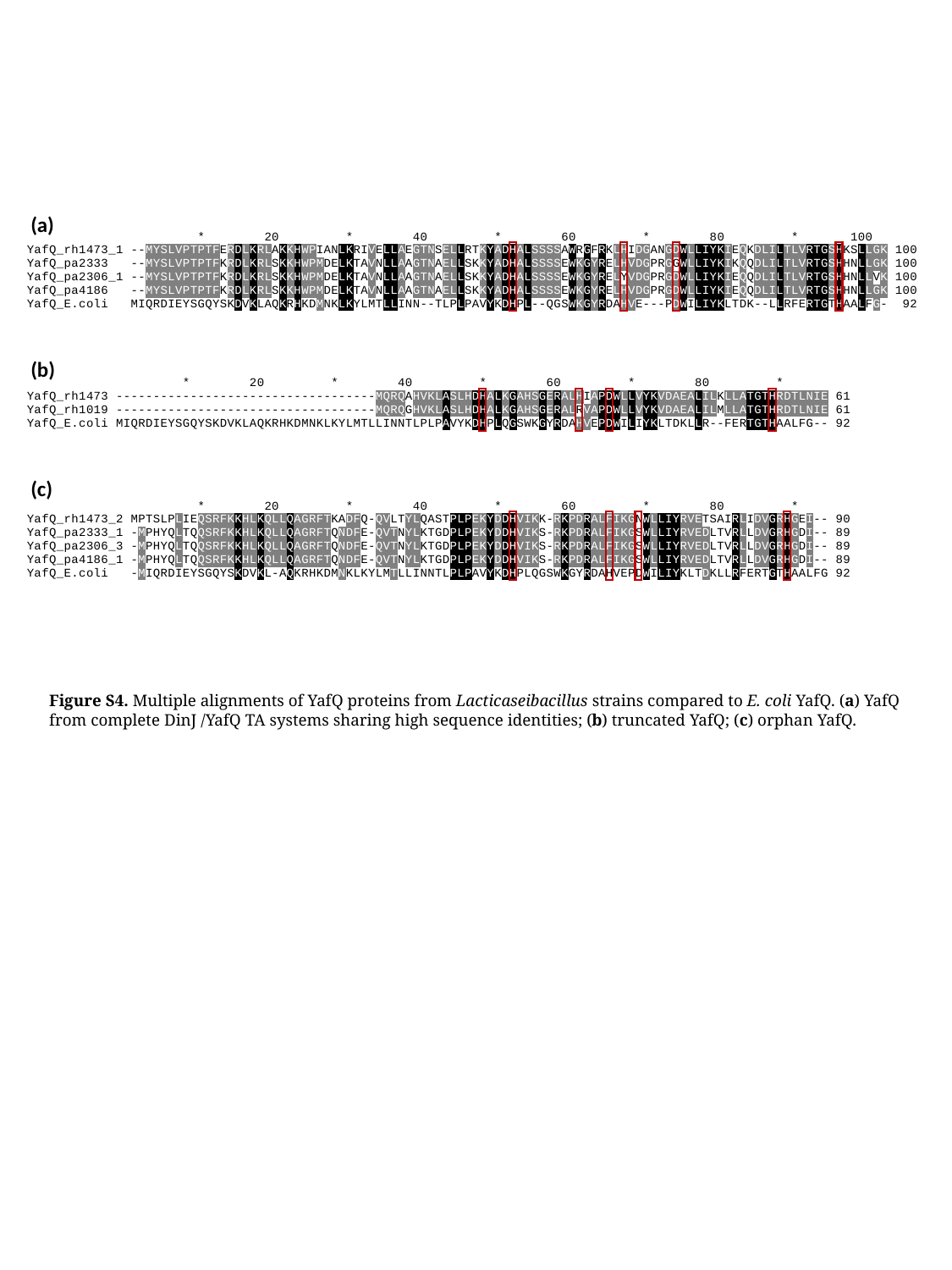

(a)
(b)
(c)
Figure S4. Multiple alignments of YafQ proteins from Lacticaseibacillus strains compared to E. coli YafQ. (a) YafQ from complete DinJ /YafQ TA systems sharing high sequence identities; (b) truncated YafQ; (c) orphan YafQ.
