# Supplementary material for: Genome Sequencing of five Lacticaseibacillus Strains and Analysis of Type I and II Toxin-Antitoxin System Distribution
Source: Microorganisms. 2021 Mar 21;9(3):648. doi: 10.3390/microorganisms9030648 (PMC8003834; doi:10.3390/microorganisms9030648)
Supplement: Supplementary file 1 [file microorganisms-09-00648-s001.zip › microorganisms-1130680 Suppl final/supplementary figure 5_rev.pptx]

## Slide 1
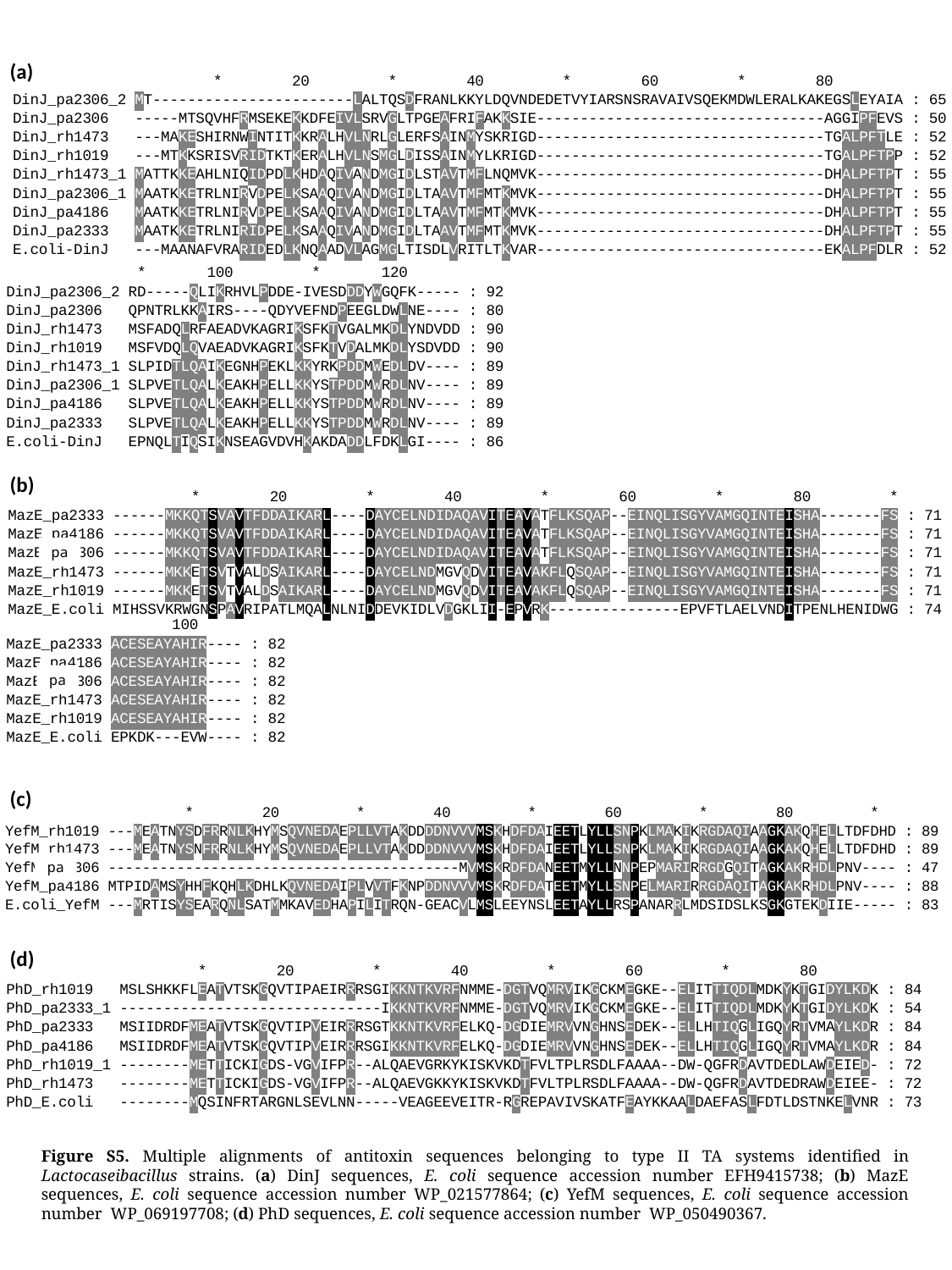

(a)
(b)
pa
pa
(c)
pa
(d)
Figure S5. Multiple alignments of antitoxin sequences belonging to type II TA systems identified in Lactocaseibacillus strains. (a) DinJ sequences, E. coli sequence accession number EFH9415738; (b) MazE sequences, E. coli sequence accession number WP_021577864; (c) YefM sequences, E. coli sequence accession number WP_069197708; (d) PhD sequences, E. coli sequence accession number WP_050490367.
